# *Bacillus cereus*, a serious cause of nosocomial infections: Epidemiologic and genetic survey

**DOI:** 10.1371/journal.pone.0194346

**Published:** 2018-05-23

**Authors:** Benjamin Glasset, Sabine Herbin, Sophie A. Granier, Laurent Cavalié, Emilie Lafeuille, Cyprien Guérin, Raymond Ruimy, Florence Casagrande-Magne, Marion Levast, Nathalie Chautemps, Jean-Winoc Decousser, Laure Belotti, Isabelle Pelloux, Jerôme Robert, Anne Brisabois, Nalini Ramarao

**Affiliations:** 1 Micalis Institute, INRA, AgroParisTech, Université Paris-Saclay, Jouy-en-Josas, France; 2 Université Paris-Est, Anses, Laboratory for Food Safety, Maisons-Alfort, France; 3 CHU Toulouse, Service de Bactériologie-Hygiène, IRSD, Université de Toulouse, INSERM, INRA, ENVT, UPS, Toulouse, France; 4 Sorbonne Universités, UPMC Univ Paris 06, Inserm, U1135, Centre d’Immunologie et des Maladies Infectieuses (CIMI-Paris), Paris, France; 5 Laboratoire de Bactériologie-Hygiène, Hôpitaux Universitaires Pitié-Salpêtrière-Charles Foix, APHP, Paris, France; 6 MaiAGE, INRA, AgroParisTech, Université Paris-Saclay, Jouy-en-Josas, France; 7 CHU Nice, Laboratoire de bactériologie, Nice, France; 8 Hôpital de Chambéry, Laboratoire de Biologie Médicale, Chambéry, France; 9 Hôpitaux Universitaires Paris-Sud Antoine Béclère, Laboratoire Hygiène, Clamart, France; 10 CHU Strasbourg, Laboratoire d’hygiène hospitalière, Strasbourg, France; 11 CHU Grenoble, Laboratoire de Bactériologie, Grenoble, France; University of Texas Medical School at Houston, UNITED STATES

## Abstract

*Bacillus cereus* is the 2^nd^ most frequent bacterial agent responsible for food-borne outbreaks in France and the 3^rd^ in Europe. In addition, local and systemic infections have been reported, mainly describing individual cases or single hospital setting. The real incidence of such infection is unknown and information on genetic and phenotypic characteristics of the incriminated strains is generally scarce. We performed an extensive study of *B*. *cereus* strains isolated from patients and hospital environments from nine hospitals during a 5-year study, giving an overview of the consequences, sources and pathogenic patterns of *B*. *cereus* clinical infections. We demonstrated the occurrence of several hospital-cross-contaminations. Identical *B*. *cereus* strains were recovered from different patients and hospital environments for up to 2 years. We also clearly revealed the occurrence of inter hospital contaminations by the same strain. These cases represent the first documented events of nosocomial epidemy by *B*. *cereus* responsible for intra and inter hospitals contaminations. Indeed, contamination of different patients with the same strain of *B*. *cereus* was so far never shown. In addition, we propose a scheme for the characterization of *B*. *cereus* based on biochemical properties and genetic identification and highlight that main genetic signatures may carry a high pathogenic potential. Moreover, the characterization of antibiotic resistance shows an acquired resistance phenotype for rifampicin. This may provide indication to adjust the antibiotic treatment and care of patients.

## Introduction

*Bacillus cereus* is a spore forming and ubiquitous bacterium present in soil, foods, insect larvae, almost all surfaces and human skin [1, 2]. Besides food poisoning [3, 4], *B*. *cereus* induces local and systemic infections [5–13]. The main described conditions are septicemia, endophthalmitis, pneumonia, endocarditis, meningititis and encephalitis, especially in immunosuppressed individuals such as neonates, resulting in the patient death in about 10% of cases [9, 14–19]. In addition, several cases of fulminant infections similar to anthrax, and affecting healthy persons, have also been reported [20–22]. Predisposing factors include intravenous drug use, surgical or traumatic wounds, intravascular catheters and prematurity due to an immature immune response and to the presence of indwelling devices in the intensive care environment of neonates [16–18, 23]. Environmental reservoirs include air filtration/ventilation equipment, linen, medical devices and hands of the staff [9, 24]. Case reports describe mainly individual cases or come from single hospital centers and no large survey has been done on *B*. *cereus* clinical infections. In addition, information on the genetic and phenotypic characteristics of the incriminated strains is generally scarce. An appropriate empirical antibiotic therapy should be started immediately after suspicion of *B*. *cereus* infection. However, as *B*. *cereus* is mainly considered as an environmental contaminant, delays in treatments may compromise the clinical outcome.

We performed a thorough description of clinical cases, together with an accurate phenotypic and genetic characterization of the strains. This should be of major interest to improve treatments of patients with non-gastrointestinal *B*. *cereus* infections.

## Material and methods

### Data collection

Epidemiological and clinical data on *B*. *cereus* samples isolated from patients were retrospectively collected from French voluntary hospitals between 2008 and 2012. Nine hospitals localized in different regions of France participated to this non-exhaustive study. The authors did not have access to any patient identifying information as part of this work. Each hospital filled a questionnaire and reported every cases of patient for which *B*. *cereus* was isolated in at least one biological sample. *B*. *cereus* strains were locally identified by plating on specific agar media (Mossel Medium) and confirmed by using 16S rDNA sequencing. These data allowed to classify the strains as belonging to the *B*. *cereus* group and excluded *B*. *anthracis* strains. Data included basic demographic data, hospital wards, type of clinical sample, date of sampling, clinical data, antibiotic therapy and outcome.

In addition, *B*. *cereus* strains obtained from surface samples around clinical cases were also included in the microbiological analysis.

### Biochemical analysis

All strains were tested for their capacity to hydrolyze starch on Plate Count Agar, their hemolytic activity on blood sheep agar plates and their lecithinase activity on Mossel medium as previously described [2, 25, 26].

### Molecular analysis

M13 sequence-based polymerase chain reaction (M13-PCR) is derived from an RAPD technique that allows differentiating between various strain patterns. M13 typing was performed as described [3]. The DNA profiles were analyzed with BioNumerics 7.1 software (Applied Maths). The software compared the DNA profiles and clustered the strains according to their similarity.

The toxin gene profiles were identified by assessing the presence of the *cytK-1*, *cytK-2*, *HBLA*, *HBLC*, *HBLD*, *NHEA*, *NHEB*, *NHEC*, *hlyII* and *ces* genes by PCR using specific primers [3] (S1 Fig). The strains were then clustered into genetic signatures (GS) according to their different combinations of presence/absence patterns. We noted a potential discrepancy for 2 strains isolated from the same patient (18), one of which was positive for the *ces* gene and the other one negative. Similarly, two other strains (from patients 16 and 17), which showed otherwise identical profiles, were negative for the *ces* gene. This might reflect a non recognition of the primers for these specific strains.

The strains were affiliated to the seven known phylogenetic groups according to the partial sequencing of the *panC* gene [27]. The complete sequence of the *panC* gene was used to compare the strains in the same cluster.

### Genomic divergence estimation

Genomes from eight bacterial isolates were sequenced using Illumina NextSeq500 paired-end 100 bp sequencing technology with 12.5 million reads per sample. For the purpose of SNP calling, *B*. *cereus* ATCC 10987 (NC_003909.8) was selected as a reference after computing Average Nucleotide Identity (ANI) (http://enve-omics.ce.gatech.edu/ani/) between preliminary de novo assemblies for the eight samples and several complete genomes available in the public databases (~95% ANI between NC_003909.8 and our samples, which is comparable to the divergence between our samples). Sequencing adaptors were removed from the reads using *Cutadapt* (version 1.8.3; with options *-n 5 -O 3 -m 0* options). Low quality sequence data were trimmed using *Sickle* (https://github.com/najoshi/sickle) (*-n -q 20 -l 2*0). Read mapping on the reference was performed using *Bwa mem* (http://bio-bwa.sourceforge.net/bwa.shtml) (v0.7.12-r1039; default options). Mapping depth excluding multiple mapped reads was extracted using *Samtools* (v1.2) *depth* (*-Q 1*) and the core genome was defined as positions with mapping depth > = 10 in the eight samples (which represented 3463800 bp). SNP calling was performed using *Samtools mpileup* and *Bcftools call* (*-vmO v*) and variants with a quality above 250 were selected. Pairwise divergence between samples was calculated as the proportion of variable positions along the core genome. A tree depicting the relationships between our eight samples was obtained by hierarchical clustering based on the matrix of pairwise divergences using R (http://www.R-project.org/) function *hclust*.

All raw reads generated were submitted to the European Nucleotide Archive (http://www.ebi.ac.uk/ena/) under the study accession number PRJEB18787.

### Toxin production

The production of the enterotoxins NHE and HBL was tested with the immunological tests BCET-RPLA Toxin Detection (Oxoïd) and Tecra (BDE VIA, 3M-Tecra) kits, respectively [28]. The production of NHE enterotoxin was semi-quantitatively assessed. According to the values obtained and following the manufacturer recommendation, the NHE production was scored as high (4–5), medium/weak (2–3) or not detectable (0–1). The production of HBL enterotoxin was quantitatively assessed and scored according to the dilution showing an activity as highly producer (1/64-1/32-1/16) or medium/weak producer (1/8-1/2).

### Antibiotic susceptibility

The Minimum Inhibitory Concentrations (MICs) of selected antimicrobial agents were measured by using concentration gradient strips (Etest^®^, BioMerieux). Briefly, inoculum was adjusted to 0.5 McFarland before being swabbed on a Mueller-Hinton agar plate (Bio-Rad). Incubation was performed at 35°C for 16–18 hours. The following agents were tested: ampicillin^$^, cefotaxime, imipenem^$^, vancomycin^$^, gentamicin^$^, rifampicin^$^, tetracycline^$^, ciprofloxacin^$^, chloramphenicol^$^, azithromycin, sulfamethoxazole/trimethoprim^$^ and clindamycin^$^. Due to scarce availability of interpretative criteria in the literature, clinical breakpoints were used when available (^$^) [29].

### Molecular typing and statistical analysis

**Fig 1:**
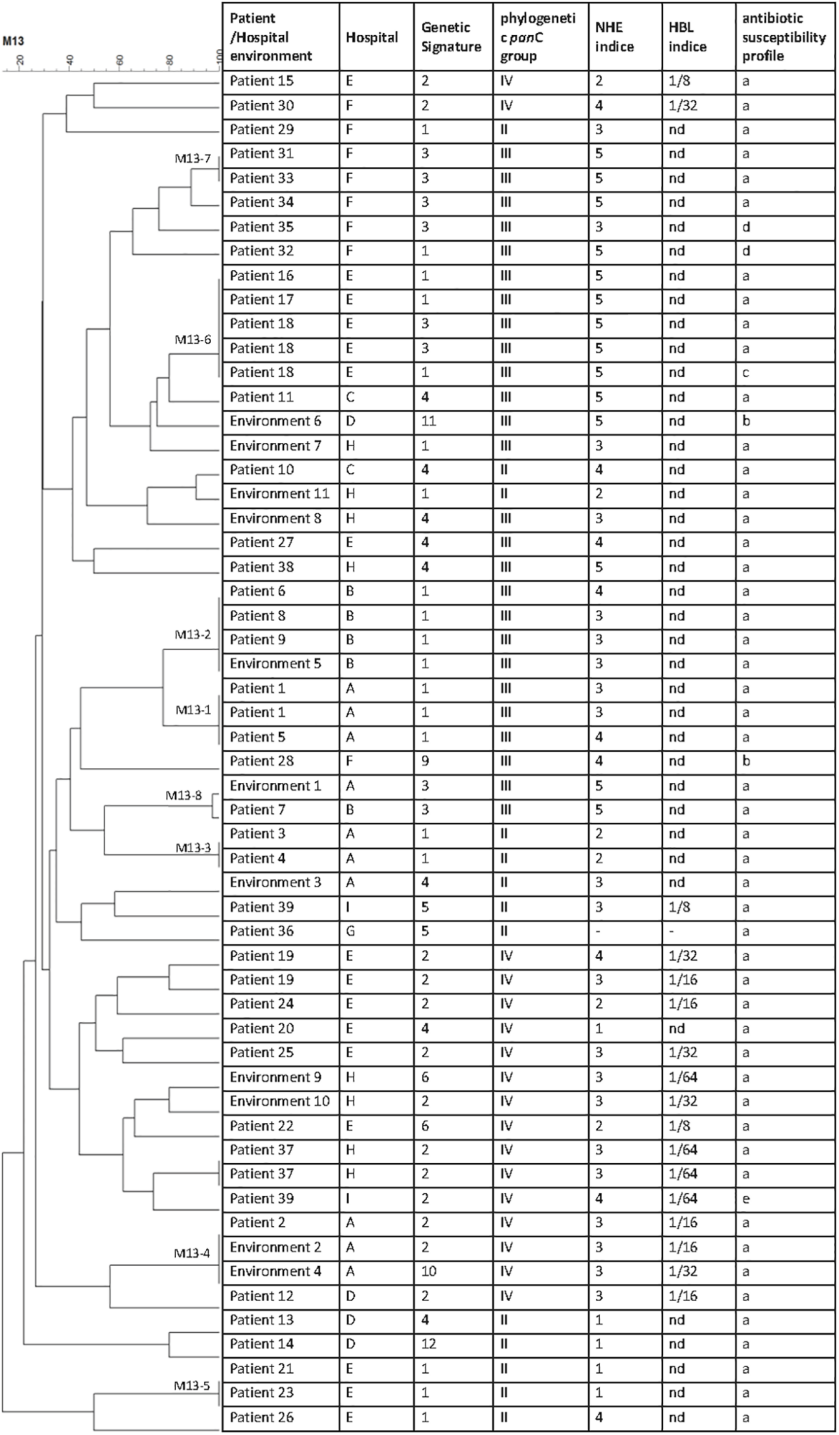
Dendogram and strain data. Left panel, dendrogram obtained by cluster analysis of M13-PCR fingerprint patterns of the 56 strains. The UPGMA was used to build a dendrogram from a pair wise similarity matrix. Seven clusters were obtained with strains sharing 100% of homology. Right panel, data include for each strain the corresponding patient and hospital, genetic signature, phylogenetic *pan*C group, NHE and HBL indice, and the antibiotic susceptibility profile. nd: not detected.

For each strain, all results were entered into a central database using BioNumerics (BN) software. A phylogenetic tree and a dendrogram from pairwise similarity matrices were built based on *panC* sequence alignments and M13-PCR molecular typing, respectively using UPGMA (Unweighted Pair Group Method with Arithmetic Mean). The percentage of identity between strains corresponding to the mean of the three experiments was used to construct the dendogram of Fig 1.

Data were analyzed by the FactoMineR package of the R 2.13.0 software (http://www.agrocampus-ouest.fr/math/). Principal Component Analysis (PCA) transforms a set of putative correlated variables into new variables, which are mutually orthogonal (uncorrelated) linear combinations of the original variables. These new variables are called principal components (PC). Each PC is defined by the coefficients in the linear combination of the original variables. For PCA, quantitative values were used for toxin production and age of patients. Each patient’s isolate was represented by a point whose coordinates corresponded to the scores contributing to the PC. The variable corresponding to the different Genetic Signatures was considered as qualitative variable. A hierarchical clustering was performed on the PC (HCPC function of FactoMineR package), in order to identify subsets of objects that corresponded to clusters having similar characteristics within the whole collection.

Simpson index of discrimination (D) was calculated according to the equation provided in [30], where N represents the total number of strains (N = 56) and n_j_ represents the number of strains belonging to each typing sub-group.

## Results

### Epidemiology

Nine hospitals reported 39 patients with *B*. *cereus* strains isolated in at least one clinical sample during the five-year study period. For the microbiological analysis, a single *B*. *cereus* strain was included per patient, except if several strains were isolated in different clinical sites (patient 1) or over a prolonged time period (patients 18 and 19). It resulted that 45 *B*. *cereus* strains were further analyzed, in addition to 11 strains isolated from hospital surface samples (Table 1, several samples were isolated from patients 1, 18 and 19).

**Table 1:** Epidemiological and clinical data of patients and samplings.

Hospitals, n	9
Strains, n	56
Patients, n	39
Environment sample, n	11
Male patients, n (%)	23 (59%)
Immunocompromised, n (%)	23 (59%)
Death, n (%)	8 (21%)
**Age**, n (%)	
Premature newborn	12 (31%)
Newborn	4 (10%)
1–25	3 (8%)
26–59	9 (23%)
60 +	10 (26%)
Unknown	1 (2%)
**Ward**, n (%)	
Neonatology	13 (33%)
Intensive care unit	6 (15%)
Medical	5 (13%)
Hematology and Oncology	5 (13%)
Surgery	4 (10%)
Emergency room	2 (5%)
Bacteriology laboratory	2 (5%)
Mortuary	1 (3%)
Unknown	1 (3%)
**Environmental sampling**, n (%)	
Surface of neonatology ward	6 (55%)
Incubator heater	3 (27%)
Milk on gastric feeding tube	1 (9%)
Catheter for sonogram	1 (9%)

The collection contains 56 strains from nine hospitals, 45 strains isolated from 39 patients and 11 strains collected on surface samples.

A majority of strains (41%) were isolated in newborns, among which 3/4 were premature infants with low birth weight. Patients over 60 year old represented the second most frequent group of patients (26%), followed by middle aged patients (23%). Wards of hospitalization and symptoms recorded were diverse (Tables 1 and 2).

**Table 2:** Characteristics of patients or hospital environment displaying *B*. *cereus* positive samples.

Sampling	Hospital	Date of sampling	Hospital ward	Age of patient	Type of sampling	Symptoms	Antibiotic treatment	Outcome
Patient 1	Hospital A	28/07/2009	neonatology	Premature newborn	blood culture	meningitis, infection in the liver, both lungs		death
Patient 1	Hospital A	28/07/2009	neonatology	Premature newborn	cerebrospinal fluid	meningitis, infection in the liver, both lungs		death
Patient 2	Hospital A	16/06/2009	neonatology	Premature newborn	blood culture	brain abscess	VAN, CTX	recovery
Patient 3	Hospital A	05/07/2009	neonatology	Premature newborn	blood culture	bacteremia	VAN	recovery
Patient 4	Hospital A	30/06/2009	neonatology	newborn	neonatal gastric liquid	bacteremia	VAN	recovery
Patient 5	Hospital A	21/07/2009	neonatology	newborn	Umbilical	local colonization	CTX, AMX, AMK	recovery
Hospital environment 1	Hospital A	23/07/2009	neonatology		Surface of neonatology ward (Window sill)			
Hospital environment 2	Hospital A	23/07/2009	neonatology		Surface of neonatology ward (Window sill)			
Hospital environment 3	Hospital A	30/07/2009	neonatology		Surface of neonatology ward (Delivery room)			
Hospital environment 4	Hospital A	04/08/2009	neonatology		Surface of neonatology ward (air vent)			
Patient 6	Hospital B	03/09/2009	neonatology	newborn	axilla later feces	skin infection	CRO	recovery
Patient 7	Hospital B	17/09/2009	neonatology	premature newborn	stomach tube feeding	premature birth	CTX, AMK, AMX (3 days)	recovery
Patient 8	Hospital B	20/09/2009	neonatology	premature newborn	gastric acid	neonatal infection	VAN (7days)	recovery
Patient 9	Hospital B	21/09/2009	neonatology	premature newborn	central venous catheter	bacteremia	AMX, AMK (3 days), then VAN (18 days)	recovery
Hospital environment 5	Hospital B	22/09/2009	neonatology		Surface of neonatology ward			
Patient 10	Hospital C	02/08/2011	neonatology	premature newborn	blood culture	refractory hypoxemia, chronic bronchial dysplasia, stage-ii intraventricular hemorrhage, sepsis	CTX, VAN, AMK (10 days)	recovery
Patient 11	Hospital C	08/2011	neonatology	premature newborn	blood culture	apnea, bradycardia, and gray complexion. after that, sepsis, organ failure and pulmonary and cerebral abscesses [18]		Death
Hospital environment 6	Hospital D		neonatology		milk on stomach tube feeding			
Patient 12	Hospital D	06/2009	emergency	80	Thoracentesis	pulmonary infection	AMX	
Patient 13	Hospital D	12/2010	neonatology	premature newborn	stomach tube feeding	abdominal distension followed by severe enterocolitis and biological abnormalities [17]	VAN, CTX, MTZ	recovery
Patient 14	Hospital D	12/2010	neonatology	premature newborn	stomach tube feeding	abdominal distension appeared three days after birth associated with radiologic, clinical, and biologic signs of enterocolitis	VAN, CTX, MTZ	recovery
Patient 15	Hospital E	18/09/2011	intensive care unit of Tropical and Infectious Diseases	30	blood culture	endocarditis associated to methicillin- sensitive Staphylococcus aureus (MSSA) in an intravenous drug abuser, and cerebral mycotic aneurysms	GEN, OXA (4 days)	death
Patient 16	Hospital E	02/11/2009	hematology	65	blood culture	sepsis causing death in a very pejorative context (leukocytes 0.3, platelets 20)		death
Patient 17	Hospital E	12/09/2011	nephrology	54	blood culture	sepsis and undernourishment	VAN, CRO, then VAN, CIP (21 days)	recovery
Patient 18	Hospital E	03/03/2010	gastroenterology	63	blood culture	bacteremia and central venous catheter-linked infection	AMX, then CIP (21 days), then GEN (3days), IPM (18days), then CIP, VAN (10 days)	recovery
Patient 18	Hospital E	26/03/2010	gastroenterology	63	blood culture	bacteremia and central venous catheter-linked infection	AMX, then CIP (21 days), then GEN (3days), IPM (18days), then CIP, VAN (10 days)	recovery
Patient 18	Hospital E	27/05/2010	gastroenterology	63	blood culture	bacteremia and central venous catheter-linked infection	AMX, then CIP (21 days), then GEN (3days), IPM (18days), then CIP, VAN (10 days)	recovery
Patient 19	Hospital E	01/12/2010	hematology	61	blood culture	sepsis (patient with an acute myeloid leukemia)	PIP, AMK, VAN (7 days), then CIP, GEN	recovery
Patient 19	Hospital E	07/12/2010	hematology	61	blood culture	sepsis (patient with an acute myeloid leukemia)	PIP, AMK, VAN (7 days), then CIP, GEN	recovery
Patient 20	Hospital E	03/06/2008	surgery	34	blood culture	bacteremia (drug addict patient with axillary abscess)		recovery
Patient 21	Hospital E	27/11/2010	neurology	newborn	blood culture	kidneys and urinary infections	CRO, GEN	recovery
Patient 22	Hospital E	15/06/2008	neurology	43	blood culture	bacteremia		recovery
Patient 23	Hospital E	06/10/2009	oncology	66	blood culture	bacteremia (patient with a colorectal cancer)		recovery
Patient 24	Hospital E	24/09/2010	hematology	24	blood culture+ skin infection	sepsis and aplastic anemia caused by drugs	PIP, AMK	recovery
Patient 25	Hospital E	12/08/2009	gynecological surgery	77	blood culture	bacteremia (patient with breast cancer)	CIP	recovery
Patient 26	Hospital E	16/07/2010	cardiac surgery	60	blood culture	sternum abscess, absent fever		Sequela of osteitis
Patient 27	Hospital E	20/06/2008	hematology	40	blood culture	bacteremia (immunocompromised patient)		recovery
Patient 28	Hospital F	07/2011	orthopedic surgery	31	Prosthesis from tibia	no clinical sign of infection	AMX	recovery
Patient 29	Hospital F	10/2011	intensive care unit	76	blood culture	community acquired pneumonia	CTX, SPI then CTX	recovery
Patient 30	Hospital F	09/2012	intensive care unit	46	catheter culture without an blood positive culture	heart failure and multiple infectious episodes	VAN, CLO, GEN then AMX then PIP then IPM then IPM, CAZ, CIP	recovery
Patient 31	Hospital F	09/2012	intensive care unit	48	blood culture	acute respiratory distress syndrome	CRO, GEN then CAZ, then PIP then CAZ, VAN, AMK	recovery
Patient 32	Hospital F	06/2011	intensive care unit	86	blood culture from catheter	heart failure, ventilator-associated pneumonia, ischemic stroke	AMK, IPM then IPM	recovery
Patient 33	Hospital F	10/2011	emergency	24	blood culture	abdominal pain, shivering, vomiting, fever, diarrhea	none	recovery
Patient 34	Hospital F	10/2012	intensive care unit	56	blood culture from catheter	bronchogenic carcinoma, pneumonia	CTX then PIP then AMK, IPM	death
Patient 35	Hospital F	09/2012	gastroenterology	85	Liver abscess	sepsis, hepatitis c and liver abscess, abdominal pain, diarrhea	GEN, CTX, then CTX, CIP, then SXT, OFX, CTX	recovery
Patient 36	Hospital G	09/2013		?	blood culture	nausea, abdominal pain and vomiting		?
Hospital environment 7	Hospital H		clinical laboratory		babies environment			
Hospital environment 8	Hospital H		clinical laboratory		environment of incubator heater			
Hospital environment 9	Hospital H		clinical laboratory		Incubator environment			
Hospital environment 10	Hospital H		clinical laboratory		Catheter for sonogram			
Hospital environment 11	Hospital H		clinical laboratory		Incubator environment			
Patient 37	Hospital H	12/2013	clinical laboratory	Premature newborn	Blood culture from umbilical venous catheter	septic shock, multiple organ failure, pulmonary and cerebral abscesses	VAN	death
Patient 37	Hospital H	12/2013	clinical laboratory	Premature newborn	blood culture from peripheral veins	septic shock, multiple organ failure, pulmonary and cerebral abscesses	VAN	death
Patient 38	Hospital H	12/2013	clinical laboratory	Premature newborn	Bronchial aspiration (lung)	septic shock and pneumonia pulmonary necrotic abscesses, recurrent pneumothorax	VAN	death
Patient 39	Hospital I	2014		?	Biopsy (kidney)	vomiting and diarrhea		death
Patient 39	Hospital I	2014		?	Biopsy (spleen)	vomiting and diarrhea		death

Data included hospital wards, date of sampling, patient age, type of sample, infection sites, clinical data, antibiotic therapy and outcome.

CTX: cefotaxime, VAN: vancomycin; AMK: amikacin; AMX: amoxicillin; MTZ: metronidazole; OXA: oxacillin; CRO: ceftriaxone; CIP: ciprofloxacin; IPM: imipenem; PIP: piperacillin; CAZ: ceftazidime; CLO: cloxacillin; SXT: cotrimoxazole; OFX: ofloxacin; GEN: gentamicin; SPI: spiramycin

*B*. *cereus* infections led to local and systemic infections (Table 2). Local infections represented 8% of the cases. A total of 28 (72%) patients had a positive blood culture for *B*. *cereus*. Among them, 15 had another body site displaying *B*. *cereus* including the lungs (n = 7) or the central nervous system (n = 5). The gastrointestinal tract represented 18% of the clinical sites. 15% patients had at least three clinical sites positive with *B*. *cereus*. Death occurred in eight (21%) patients, including four premature babies.

It is noteworthy that, for 62% (n = 24) of patients, *B*. *cereus* was considered as the potential cause of infections and usually taken into account by the physicians for the antibiotic therapy. In the remaining cases, *B*. *cereus* was initially wrongly considered as a contaminant.

### Biochemical identification

Among the isolates characterized as *B*. *cereus* group strains, 48% presented the ability to hydrolyze starch, 93% had lecithinase activity and 71% were hemolytic. These data are consistent with previous finding showing that not all *B*. *cereus* strains are hemolytic [3]. The production of two main toxins, NHE and HBL, was assessed. 25% of the strains were high producers of NHE and high producers of HBL, 54% were high producers of NHE and low or no producers of HBL, 7% were low producers of NHE and high producers of HBL, and 14% were low or no producers of NHE and HBL (Fig 1).

### Molecular characterization

The presence of ten genes suspected or shown to play a role during *B*. *cereus* pathogenesis was investigated. The combination of these genes allowed clustering the strains into ten genetic signatures (GS) **(**S2 Fig). The *cytK1* gene was not found in the strain collection.

The most frequent GS were GS1 (*nhe* only, 34%), GS2 (*nhe*, *hbl*, *cytk-2*, 23%), GS3 (*nhe*, *ces*, 16%) and GS4 (*nhe*, *cytk-2*, 14%) (Fig 1).

Our clinical strains belonged to only three phylogenetic groups by *panC* sequence analysis II, III and IV representing 23%, 47% and 30% of the strains, respectively (Fig 1).

Finally, the M13 pattern of each strain was assessed. A dendogram from pair wise similarity matrixes was build based on M13-PCR molecular typing using UPGMA (Unweighted Pair Group Method with Arithmetic Mean). 41 different M13 profiles were identified according to the percentage of identity between strains (Fig 1). The discriminating Simpson’s index revealed that M13 PCR allowed a high power of differentiation of the strains (discrimination index 0.983).

### Antimicrobial susceptibility

**Fig 2:**
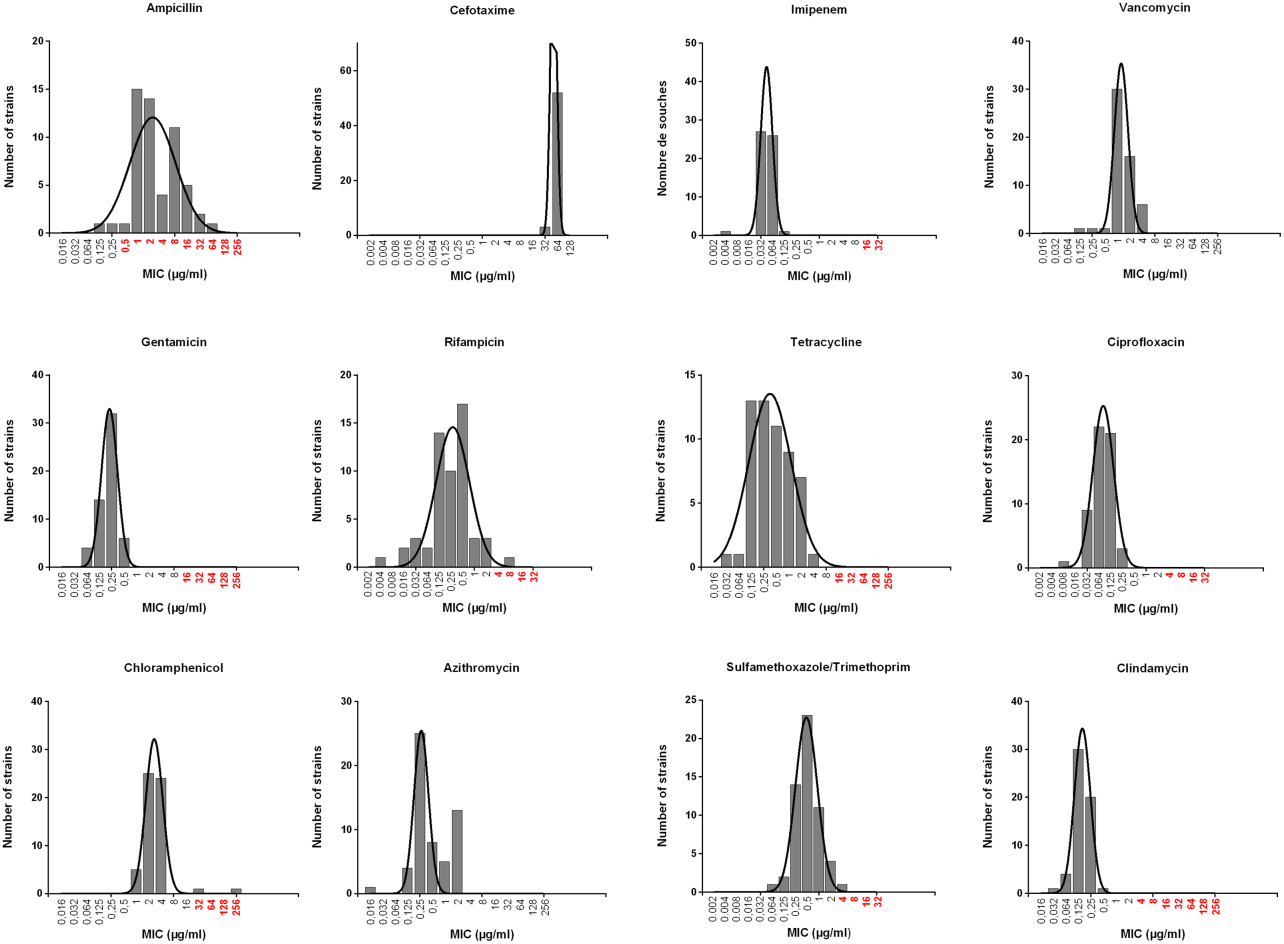
MIC results (Etest method) for the 56 *B*. *cereus* strains. Black lines: population distribution. Concentrations indicated in red are classified as clinically resistant according to CLSI or EUCAST (no known values for Azithromycin, cefotaxime and vancomycin).

The Minimum Inhibitory Concentrations (MICs) of selected antimicrobial agents were measured. According to CLSI clinical breakpoints, the tests revealed five susceptibility patterns (Figs 1 and 2 and S3 Fig). Natural resistance to beta-lactams was confirmed for ampicillin and cefotaxime, while imipenem appeared active at low concentrations. All strains were categorized as susceptible to vancomycin, gentamicin, tetracyclin, ciprofloxacin, azithromycin and clindamycin. Two strains were resistant to chloramphenicol. One strain was resistant to rifampicin and one to cotrimoxazole (trimethoprim/sulfamethoxazole), respectively.

### Principal component analyses

**Fig 3:**
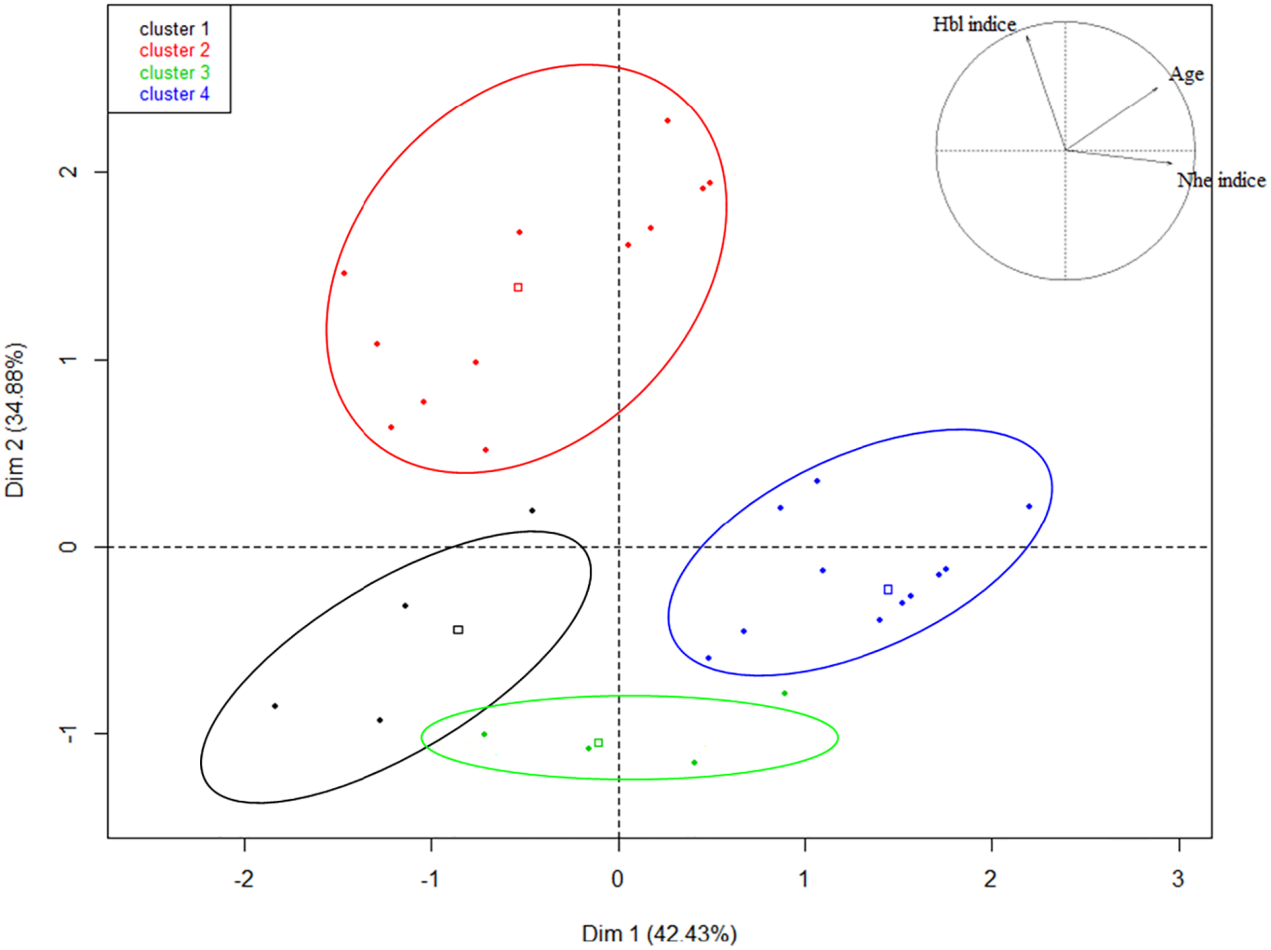
Correlation clusters of the quantitative variables characterizing each *B*. *cereus* strain isolated from patients. The percentages of variation explained by the principal components (PC1 and PC2) are indicated in brackets. The factors involved in PC1 (Dim1: age of patients and NHE indice) and PC2 (Dim2: HBL indice) are indicated in the variable factor map at the top right of the figure. The strains located inside a colored circle belong to the same cluster, as determined by the hierarchical cluster analysis performed after PCA. Each dot corresponds to a strain. The squares represent the representative value for the cluster.

To analyze the potential correlations between the phenotypic and genotypic characterizations of the strains, principal component analyses were performed for each characteristic. There were no obvious correlations between GS and/or symptoms, hospital wards and patient age. By contrast, several clusters of strains appeared when considering in the PCA the three components: age of patient, NHE production and HBL production (Fig 3). The circles of correlations indicate the strains that can be statistically grouped. Strains producing high level of HBL were on average weak producers of NHE (strains clustering in the red circle). Strains producing high level of NHE infected middle age or elderly patients (>34 year old) (blue circle). There was no correlation with strains weakly producers of HBL and NHE production or age of patients (green circle). The most striking results was the correlation between the low age of the patient (<6.5 year old) and no or weak production of HBL and NHE (strains in the black circle).

### Molecular epidemiology

#### Intra-hospital contaminations

**Fig 4:**
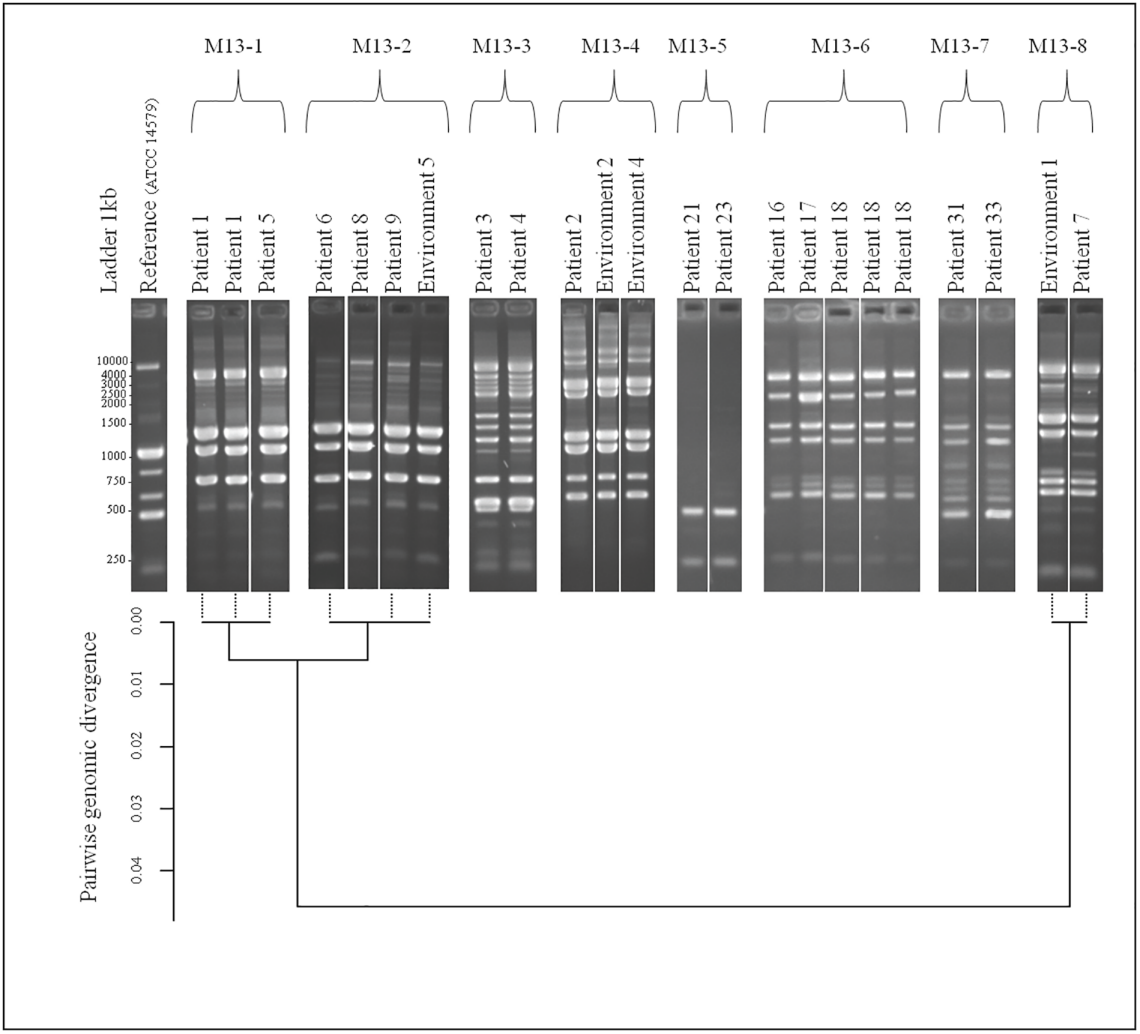
M13-PCR fingerprint patterns of *B*. *cereus* strains showing eight possible cross contaminations between patients/patients or patients/environment. Top panel, lane 1: 1kb DNA ladder. Lane 2: reference strain *B*. *cereus* ATCC14579. Lane 2 to 23: *B*. *cereus* strains. Bottom: divergence tree between eight samples obtained by hierarchical clustering based on the matrix of pairwise divergences after WGS data.

We identified eight cases of intra-hospital cross infections: the occurrence of an infection by a single strain of *B*. *cereus* of at least two different patients within the same hospital. Strains with identical M13 pattern (100% identity by UPGMA on dendogram), *pan*C sequencing (over the entire gene), GS, toxin production and antibiotic susceptibility/resistance pattern were grouped in a single molecular profile (Fig 1). Strains with identical molecular profiles were recovered from different patients and hospitals strongly suggesting the occurrence of eight hospital cross-infections. Fig 4 shows the M13 profiles (top level) and the WGS-based pairwise divergence (bottom) of the strains involved in the 8 cases of cross-infections. For M13 profiles, Fig 4 (top) shows one representative image but the calculated % of identity between strains is shown on the dendogram of Fig 1.

At hospital (A), a *B*. *cereus* strain was isolated from the umbilical cord of a newborn (patient 5). Two *B*. *cereus* isolates with identical profiles were isolated one week later from two clinical samples (blood culture and cerebrospinal fluid) from another neonate (patient 1). Patient 1 died at day 6 following a sepsis, and multiple-site infection with no antibiotherapy. The three isolates of patients 1 and 5 were characterized by the M13-1 profile, GS1, *pan*C group III, 100% identity over the *pan*C gene sequence, a medium NHE and low HBL production and the antibiotic profile a. All these similarities, and especially the M13 pattern, strongly suggested that the three isolates were identical and/or may descend from a same recent ancestor. To confirm these findings, the three genomes of the strains were entirely sequenced (WGS). The divergence tree between the strains was obtained by hierarchical clustering based on the matrix of pairwise divergences. The three genomes showed 100% identity (Fig 4 and S4 Fig). Indeed, 1 or 2 SNP differed between the strains and pairwise divergences between samples were comprised between 2.9e-07 and 8.7e-07, corresponding to 0.00%.

Therefore, the WGS data confirmed that identical strains were recovered from two unrelated patients within the same hospital. In addition, the data indicate that the genomic characterization following M13 typing allowed identifying and discriminating between strains.

At the same hospital (A), a *B*. *cereus* strain was isolated from a newborn (patient 4) and one week later, a *B*. *cereus* strain with a similar profile was isolated from another premature newborn with clinical sepsis (patient 3), constituting a second strain cluster.

Similarly, at the same hospital (A), a *B*. *cereus* strain was isolated from a premature newborn with a clinical sepsis and brain abscesses (patient 2). A strain with similar profile was isolated later twice from the hospital environment (environment 2 and 4), constituting a third cluster.

At hospital (B), a *B*. *cereus* strain was isolated from three newborns (patients 6, 8 and 9) and from an environmental sample (environment 5) during the same month. Patient 6 had local *B*. *cereus* colonization at the point of entry of catheter. The patient was treated with ceftriaxone and the catheter was removed. Patient 8 had a contaminated gastric acid and patient 9 had a bacteremia. They both received antibiotics including vancomycin and had favorable outcomes.

At hospital (E), strains with identical profiles were recovered from two patients over a one year time interval and in different hospital wards (oncology and neurology). Patient 23 was 66-year old and diagnosed with colorectal cancer and sepsis. *B*. *cereus* was isolated from a blood culture, displaying also a coagulase-negative Staphylococcus. *B*. *cereus* was therefore neglected. However, a strain with identical profile was isolated one year later from a newborn (patient 21) leading to kidney and urinary infections.

At the same hospital (E), *B*. *cereus* strains with similar profiles were recovered from three different patients over a 2-year period. The three patients were hospitalized in different wards: hematology, nephrology and gastroenterology. Patient 16, 65 year old had a positive blood culture and died without antibiotic treatment. Patient 17, age 54 had a bacteremia at the point of entry of the catheter. He had a favorable outcome after several antibiotic courses for 21 days. Patient 18, age 63, had three positive blood cultures yielding *B*. *cereus* strains with identical profiles during three months. He had a favorable outcome following three consecutive antibiotic courses.

More recently, at hospital (F), *B*. *cereus* strains with identical profiles were isolated from two patients over a one-year period. Patient 33 was 24 year-old admitted at the emergency ward with abdominal pain, shivering, vomiting, fever and diarrhea. A blood culture was positive for this *B*. *cereus* strain. Patient 31, 48-year old, was admitted in cardiology almost one year later with clinical sepsis and acute respiratory distress. A blood culture was positive with a *B*. *cereus* with identical profile and the patient received a combined antibiotic course with a favorable outcome. The two patients do not seem to have links in anyways and were admitted at the hospital 10 months apart.

These data reveal the capacity of a given *B*. *cereus* strain to persist in the hospital and to infect several patients over a long period of time (over 2 years) and at different hospital localizations.

#### Inter hospital contaminations

A case of inter-hospital contamination was identified between the hospitals (A) and (B). A newborn from hospital (B) (patient 7) had a *B*. *cereus* strain that presented an identical profile to a strain isolated the same month from the neonatal hospital environment (environment 1) in hospital (A). As these data may reveal, to our knowledge, the first inter hospital contamination ever described for *B*. *cereus*, we decided to compare them as well by WGS. The identity between the two strains was confirmed, and the strains showed 0.00% divergence and 20 differences in SNP (S4 Fig). No direct link has been identified between the patient 7 and hospital (A).

A second case of putative inter-hospital contamination was identified within the same hospitals (A) and (B). At hospital (B), a *B*. *cereus* strain was isolated from three premature newborns (patients 6, 8 and 9) and from an environmental sample (environment 5) during the same month. Surprisingly, a *B*. *cereus* strain with very similar profile (0.01% divergence and 48–49 differences in SNP) was isolated two months earlier from two newborns (patient 1 and 5) at hospital (A). Patients 1 and 5 from hospital (A) were the first newborns of this series. During the same period, patient 6 was first admitted in hospital (A) and then transferred to hospital (B), located 15 km apart. *B*. *cereus* strains with similar profiles were then isolated in hospital (B) from patient 6, 8 and 9. According to the data, there is a possibility, that patient 6 may have been contaminated during his stay in hospital (A) and transmitted the strain to other patients in hospital (B).

Although it is not fully clear from the available evidence whether the isolates are indeed of the same origin, these situations may be to our knowledge, the first examples of inter-hospital cross-contaminations with *B*. *cereus* strains.

## Discussion

*B*. *cereus* is notoriously associated with food poisoning and eye infections [31, 32]. *B*. *cereus* also induces a multitude of other serious infections such as fulminant sepsis and devastating central nervous system infections [9, 19]. In hospital, *B*. *cereus* is however usually regarded by the physicians as an environmental contaminant. Thus, despite positive blood samples, *B*. *cereus* is seldom considered as cause of infection. Consequently, the antibiotic treatment is sometimes inadequate because of the misinterpretation of clinical and bacteriological diagnosis of *B*. *cereus* infections [33].

The aim of our study was to gain a better knowledge on the consequences, sources and pathogenic strain patterns in *B*. *cereus* clinical infections. We analyzed the correlations between epidemiology, clinical, phenotypical and molecular data in order to alert clinicians regarding the emerging threat that *B*. *cereus* can represent in hospital settings.

We reported 39 patients with *B*. *cereus* infections. This study is however not exhaustive and the number of cases is likely underestimated as clinical laboratories do not necessarily complete species identification considering *Bacillus* species as environmental contaminants.

Among the 39 patients, eight (21%) died following *B*. *cereus* systemic infections. Among them, 4 were premature newborns, 1 patient had a carcinoma, 1 patient was also infected with *S*. *aureus*, 1 patient had a low leukocyte level. The underlying condition of the last one was unknown. Consistent with previous findings [14, 18], our study confirms that the majority of patients were premature newborns, followed by elderly people. It is suspected that *B*. *cereus* from the hospital environment enter the infant bodies due to the presence of indwelling devices such as catheters. However, to our knowledge, no studies could demonstrate so far that the same *B*. *cereus* strain could be recovered from a patient and its hospital environment. The comprehensive molecular characterization of the strains from our collection allowed identifying several hospital clusters. Strains with identical profiles were isolated from different patients and/or environment samples over periods of time up to two years and from different hospital settings. This clearly suggests that the same *B*. *cereus* strain is able to persist in the hospital environment despite routine cleaning procedures and may remain a source of infection for inpatients, likely due to its ability to form spores and/or biofilms [31]. In addition, we reveal the identification of strain clusters between two hospitals demonstrating the first documented cases of inter-hospital cross-contamination by *B*. *cereus* strains. It is interesting to note that *B*. *cereus* does not constitute a clonal population [34]. As an example, compared to the available reference sequenced genomes *B*. *thuringiensis* 407 (https://www.ncbi.nlm.nih.gov/assembly/GCF_000161495.1/ BT407) and *B*. *cereus* ATCC10987 (https://www.ncbi.nlm.nih.gov/assembly/GCF_000008005.1/ ATCC10987), our strains showed 91.56% and 94.87% similarity, respectively. In this situation, our WGS data showing 99.99% and 100% identity between the hospital strains strongly suggest the strain identities.

In two cases, identical *B*. *cereus* strains were isolated from two newborns with different pathologies. In both cases, the first infant had a localized colonization and the second had a systemic infection, suggesting that the severity of symptoms probably depends on the site of infection and/or the immune status of the patient.

Surface environmental samples were analyzed only in case of infection and were restricted to the patient zone. The hands of the staff or the linen were not tested. It is therefore difficult to hypothesize on the method of contamination. Our data, suggest however, that the strains remain in the hospital environment long enough to infect inpatients up to two years following the first infection. In two cases, the second infection led to patient death.

*B*. *cereus* gastrointestinal pathogenesis are considered to be mainly due to the production of toxins such as HBL, NHE, CytK or the cereulide [28, 35–37]. The virulence factors associated with clinical non-gastrointestinal diseases are unknown, although HlyII has been shown to allow *B*. *cereus* to counteract the host immune system [38–40].

34% of the strains belonged to GS1 where only *nhe* genes were detected. Nevertheless, the production of the NHE toxin was highly variable and ranged from high to very low, suggesting that other factors may play a role during *B*. *cereus* non-gastro intestinal infections. High production of one toxin NHE or HBL was correlated with low production of the other one. Indeed, 54% of the strains were high NHE producers and low HBL producers, and only 25% of the strains were high producers of both NHE and HBL. Thus, it appears necessary to identify other unknown virulence determinants to get further insights in the pathogenic potential of *B*. *cereus* during non-gastrointestinal infections or to establish whether such infections are entirely opportunistic. It would be interesting to examine the role of the PlcR regulon, which is suspected to play a major role during gastrointestinal diseases, as well as other non-PlcR regulated toxins [35, 41, 42].

Interestingly, we observed that strains isolated from low age population were in average low toxin-producers. This suggests that newborn may be particularly sensitive to *B*. *cereus* strains, even those with low toxin production, or that other unknown factors may be responsible for newborn infections.

There is no specific recommendation for the study and interpretation of *B*. *cereus* antibiotic susceptibility in Europe. The choice of antibiotic is guided by therapeutic considerations and the search for alternatives to the treatments used for prophylaxis. Our data show homogeneity of antibiotic susceptibility pattern in the strain population, which is in favor of empiric therapy as soon as *B*. *cereus* infection is identified. The data revealed the efficacy of the association of glycopeptide and aminoglycoside or imipenem and ciprofloxacin. On the opposite, due to the natural resistance of *B*. *cereus* to most beta-lactams [33] and as confirmed by our study, penicillins and third generation cephalosporins are not recommend for treating *B*. *cereus* infections.

Of interest, patient 18 had three blood samples positive for *B*. *cereus*. The initial strains were susceptible to rifampicin and the last strain displayed resistance to rifampicin. This case strongly suggests acquired rifampicin resistance over time, and that, similarly to *S*. *aureus*, rifampicin should be used with caution to treat *B*. *cereus* infections. Whether the strain acquired resistance from another bacterium or from a mutation was not studied.

Taken together, such study gathering epidemiological and clinical data together with phenotypic and molecular characterization has, to the best of our knowledge, never been done for *B*. *cereus*. This study demonstrates the high persistence capacity of *B*. *cereus* strains in the hospital environment, leading to the reemergence of strains two years after the first isolation. Strains spread within the same hospital but also between different hospitals.

The antibiotic resistance profiles should allow to quickly adapting treatment and care of patients. In conclusion, our study highlights that *B*. *cereus* isolated from patients, especially if immunosuppressed, should not be systematically disregarded as a contaminant, and its clinical significance should be raised. Inadequate attention could delay appropriate therapy and increase the risk of severe infections and poor outcome.

## Supporting information

S1 Fig Primers. List of primers used in this study.
(TIFF)

S2 Fig Toxin gene profiles called genetic signatures (GS). The toxin gene profiling was performed according to the presence or absence of nine genes (*cytK1* was absent in all strains) associated with *B*. *cereus* pathogenesis.
(TIFF)

S3 Fig *In vitro* antibiotic susceptibility profiles. Five profiles were defined from the 56 *B*. *cereus* isolated from patients or from hospital environment. S: susceptible R: resistant.
(TIFF)

S4 Fig SNP calling and pairwise divergence. SNP calling and pairwise divergence were calculated between the samples.
(TIFF)
