# Supplementary figures and images for: Bacillus cereus, a serious cause of nosocomial infections: Epidemiologic and genetic survey
Source: PLoS One. 2018 May 23;13(5):e0194346. doi: 10.1371/journal.pone.0194346 (PMC5966241; doi:10.1371/journal.pone.0194346)

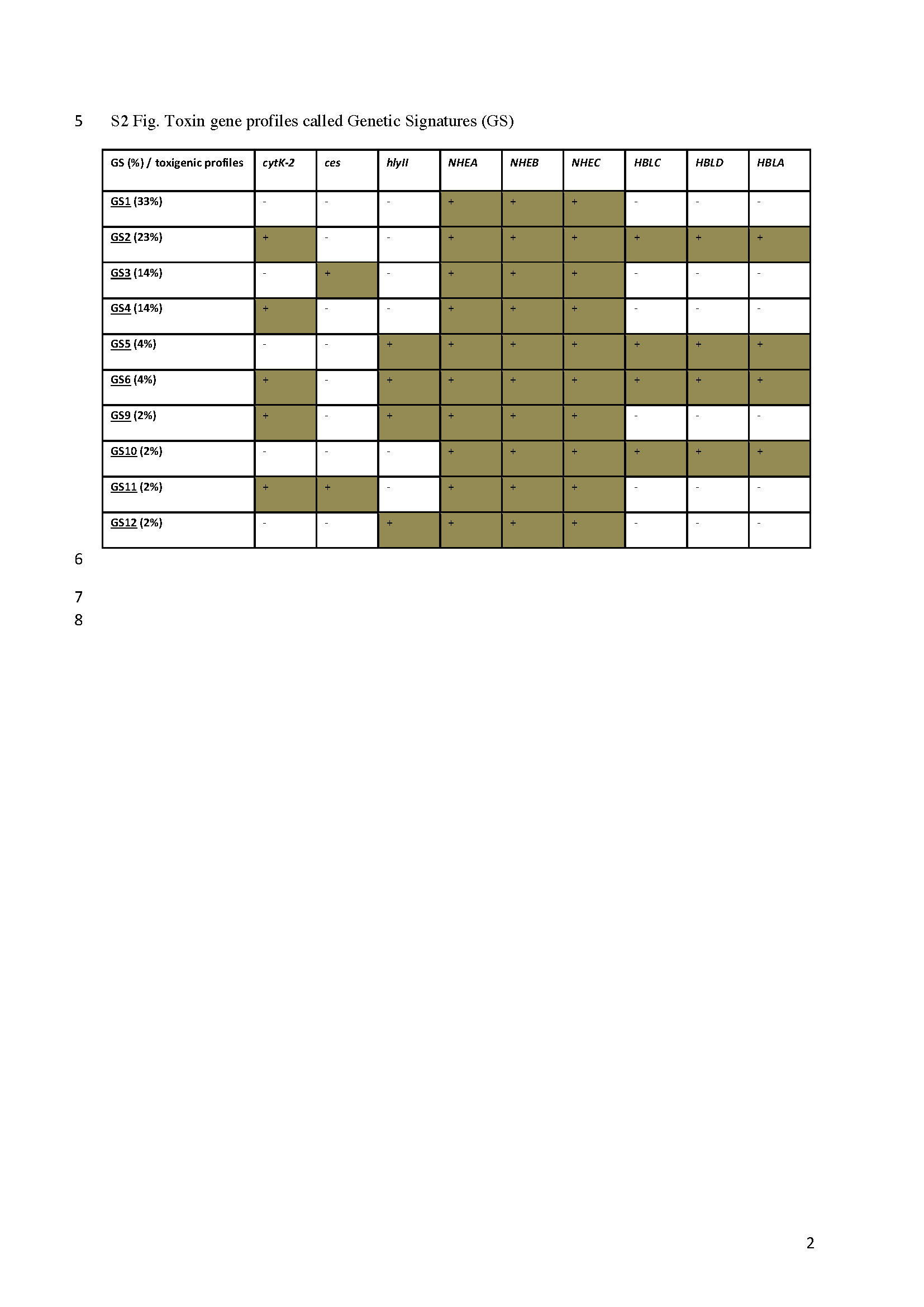

Supplement: S2 Fig — The toxin gene profiling was performed according to the presence or absence of nine genes (cytK1 was absent in all strains) associated with B. cereus pathogenesis. (TIFF) [file pone.0194346.s002.tiff]

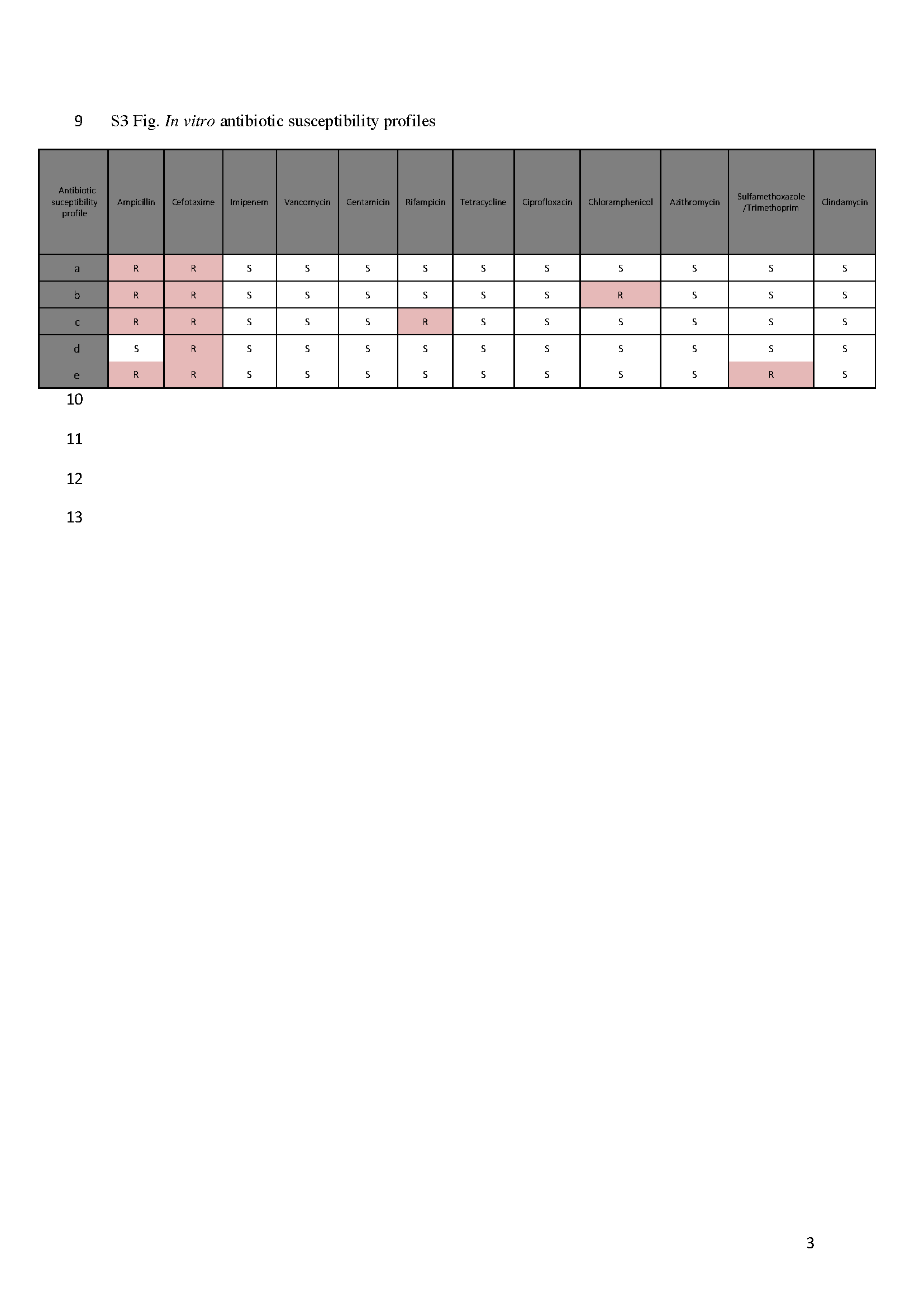

Supplement: S3 Fig — Five profiles were defined from the 56 B. cereus isolated from patients or from hospital environment. S: susceptible R: resistant. (TIFF) [file pone.0194346.s003.tiff]

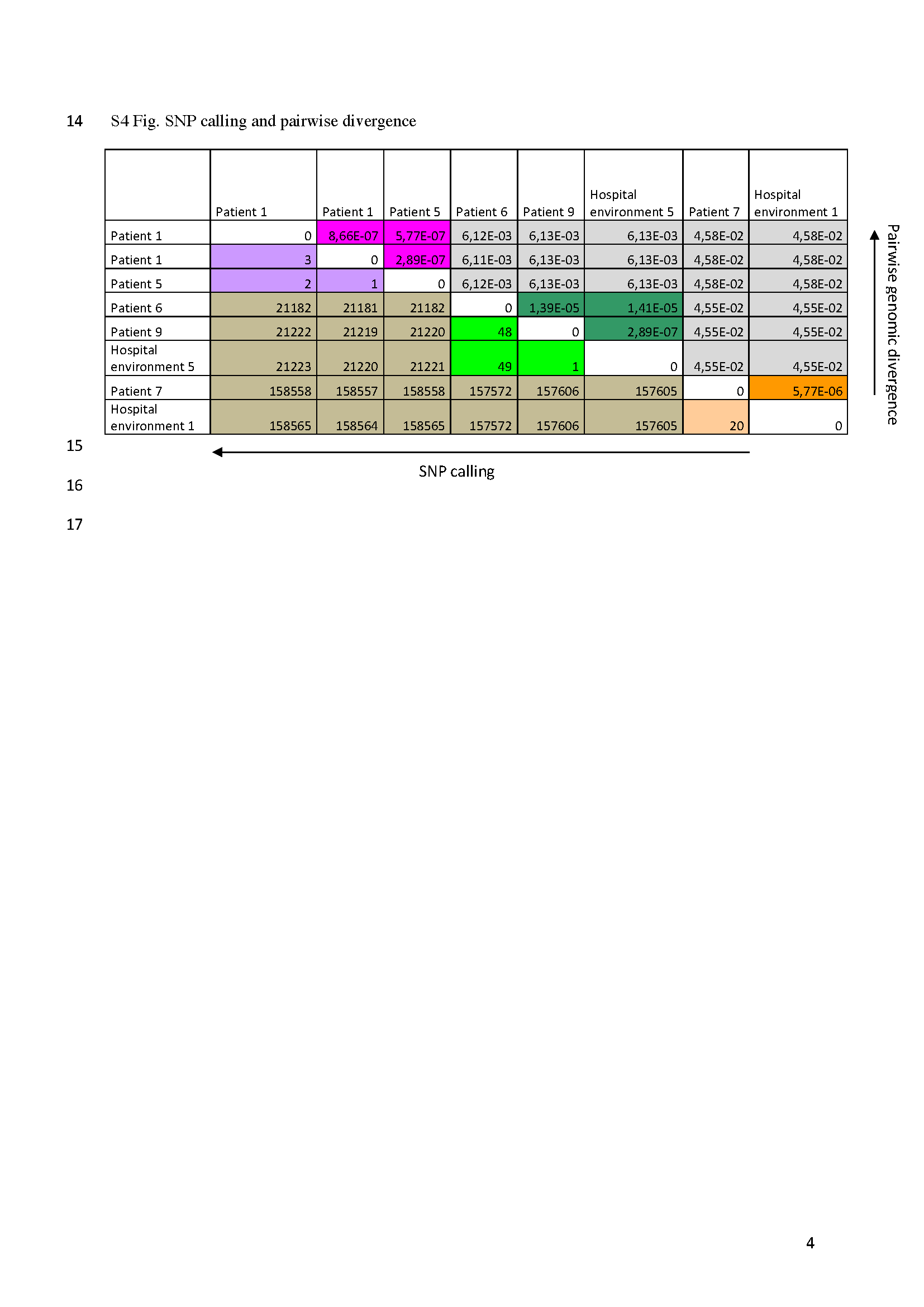

Supplement: S4 Fig — SNP calling and pairwise divergence were calculated between the samples. (TIFF) [file pone.0194346.s004.tiff]
